# Carvacrol and Thymol Modulate the Cross-Talk between TNF-*α* and IGF-1 Signaling in Radiotherapy-Induced Ovarian Failure

**DOI:** 10.1155/2019/3173745

**Published:** 2019-08-20

**Authors:** Yasmen F. Mahran, Amira M. Badr, Alhanouf Aldosari, Raghad Bin-Zaid, Hind N. Alotaibi

**Affiliations:** ^1^Department of Pharmaceutical Sciences, Faculty of Pharmacy, Princess Nourah bint Abdulrahman University, Riyadh, Saudi Arabia; ^2^Department of Pharmacology & Toxicology, Faculty of Pharmacy, Ain Shams University, Cairo, Egypt; ^3^Department of Pharmacology and Toxicology, College of Pharmacy, King Saud University, P.O. Box 22452, Riyadh 114592, Saudi Arabia; ^4^College of Pharmacy, King Saud University, P.O. Box 22452, Riyadh 114592, Saudi Arabia

## Abstract

Premature ovarian failure (POF) is a common cause of infertility in premenopausal women who are unavoidably exposed to cytotoxic therapy. Radiotherapy is one of the most effective cytotoxic treatments. However, the radiosensitivity of ovarian tissues limits its therapeutic outcome and results in the depletion of the primordial follicle and loss of fertility. Therefore, the need for an effective radioprotective therapy is evident especially when none of the current clinically used modalities for radioprotection succeeds efficiently. The present study investigated the potential radioprotective effect of carvacrol (CAR) (80 mg) or thymol (80 mg) on gamma- (*γ*-) irradiation-induced ovarian damage as well as their role in the cross-talk between IGF-1 and TNF-*α* signaling and antioxidative activity. In immature female Wister rats, a single dose of whole-body irradiation (3.2 Gy, LD_20_) produced considerable ovarian damage, which was evident by histopathological findings and hormonal changes. Interestingly, pretreatment with CAR or thymol significantly enhanced the follicular development and restored the anti-Mullerian hormone (AMH), E2, and FSH levels. Both essential oils improved the irradiation-mediated oxidative stress and reduction in proliferating cell nuclear antigen (PCNA) expression. Moreover, irradiated rats exhibited an inverse relationship between IGF-1 and TNF-*α* levels two days post irradiation, which was further inverted by the pretreatment with CAR and thymol and ought to contribute in their radioprotective mechanisms. In conclusion, CAR and thymol showed a radioprotective effect and rescued the ovarian reserve mainly through counteracting oxidative stress and the dysregulated cross-talk between IGF-1 and TNF-*α*.

## 1. Introduction

Radiotherapy is one of the most important therapies for cancer that relies on DNA damage to eradicate tumors [1]. However, this process has the unintended off-target effect of permanently damaging normal tissues that are within the treatment field [2, 3] during or shortly after the completion of irradiation and thus limiting its therapeutic outcome [4]. As the life expectancy of young cancer patients has significantly increased due to advances in cytotoxic treatments, more females of reproductive age are experiencing premature ovarian failure (POF) after radiotherapy [5], and thus, it is now considered necessary to take appropriate measures for fertility preservation in surviving cancer patients [5, 6]. The ovary is a privileged organ as it contains the female germline, and it is highly sensitive to radiation damage [7, 8]. Consequently, any factor that damages the follicle pool—such as radiation—can accelerate reproductive aging and lead to premature amenorrhea, subfertility, or even infertility [9]. In this context, radiotherapy has an array of serious effects on ovarian functions [3], such as the depletion of the nonrenewable primordial follicle reserve [10]. The estimated dose at which half of the follicles are lost in humans (LD_50_) is 4 Gy [11]. Therefore, there is an urgent need to examine the ideal prevention strategies which function to protect the entire ovarian milieu from radiation damage [12]. Some nonpharmacologic [13, 14] and pharmacologic strategies [15] have been tried in an attempt to ameliorate the gonadotoxic effect of irradiation. However, the ideal mitigation paradigm has not been discovered yet.

Exposure to ionizing radiation produces oxygen-derived free radicals and activates pathways that lead to follicular death, such as inflammatory and apoptotic pathways [12]. Furthermore, the role of insulin-like growth factor 1 (IGF-1) axis in follicular development and ovulation is well documented [16, 17]. Indeed, the IGF-1/IGF-1R axis acts as modulators of gonadotropins at the cellular level as well as stimulates granulosa cell proliferation and differentiation [18]. Besides, accumulating evidences have shown that the cross-talk of TNF-*α* and IGF-1 signaling pathways may modulate biological functions in other organs [19]. It has been demonstrated that TNF-*α* suppresses IGF-1 mRNA expression and upregulates IGF-binding protein 3 (IGFbP3) [20]. However, there is a scarcity in studies which reported on the cross-talk in ovaries or linked this to protective mechanisms in radiation-induced POF in vivo.

The use of naturally occurring compounds as a radiation modifier has become an important strategy in the field of radiotherapy. Plant-derived chemopreventive agents exhibit limited side effects and less toxicity and at the same time protect the normal cells against radiation [21]. Carvacrol (CAR) and thymol are the major monoterpenic phenols, which occur in various essential oils among plant species such as *Origanum*, *Thymus*, and *Corydothymus* [22]. It is known that CAR possesses an extensive variety of pharmacological properties including antioxidant and antimicrobial activities [23] and chemopreventive effects in 1,2-dimethylhydrazine-induced colon cancer [24]. Recent studies demonstrated that CAR suppresses the expression of inflammatory marker genes such as interleukin-4 (IL-4), interleukin-6 (IL-6), interleukin-17 (IL-17), and TNF-*α* in rats [25, 26]. Besides, some studies have shown that thymol possesses antimicrobial, antioxidant [27], and hepatoprotective effects [28]. Moreover, it markedly inhibited the production of TNF-*α* and IL-6 in lipopolysaccharide-stimulated inflammatory response in mouse mammary epithelial cells [29]. Recently, Abedi et al. reported that thymol significantly protected against acute and chronic salivary gland dysfunction induced by ionizing radiation in the rats [30]. Despite these mentioned studies, no reports explored the potential protective effects of these essential oils against radiotherapy-induced ovarian failure or linked their radioprotection in ovaries with the serum IGF-1 levels.

From the information mentioned above, the radioprotective role of CAR or thymol in vivo and the exact cellular mechanisms are not defined. Therefore, the aim of the present study was to explore the modulatory effects of CAR or thymol on radiation-induced POF in vivo as well as the possible underlying mechanisms, particularly the impact on the cross-talk between TNF-*α* and IGF-1 signaling in irradiation-induced ovarian failure.

## 2. Material and Methods

### 2.1. Drugs and Chemicals

Carvacrol (2-hydroxy-4-cymene; isothymol) and thymol (2-isopropyl-5-methylphenol) were obtained from Extrasynthese Co. (Z.I. Lyon Nord, Genay Cedex, France). Dipotassium hydrogen phosphate (K_2_HPO_4_) and potassium dihydrogen phosphate (KH_2_PO_4_) were purchased from Sigma-Aldrich (St. Louis, MO, USA). All other chemicals and solvents were of the highest grade commercially available.

### 2.2. Gamma Radiation

Animals were exposed to a single dose of whole-body gamma radiation (3.2 Gy) with a dose rate of 0.48 Gy/min using a Gamma Cell 40 biological radiator with a Cesium (^137^CS) source at the Research Centre of King Saud University, Riyadh, Saudi Arabia. This dose represents the LD_20_ according to the study of Lee et al. [31]. The plastic boxes containing rats were positioned in a chamber fixed to the irradiation equipment.

### 2.3. Animals

The study was conducted according to the ethical guidelines of the Faculty of Pharmacy, King Saud University, Saudi Arabia (IRB number; KSU-SE-19-04). Immature female rats were used at the age of 23 days (which was appropriate for this study) and were chosen according to previous studies. Animals were obtained from the animal house of the Faculty of Pharmacy, King Saud University, Riyadh, Saudi Arabia. For acclimation, rats were housed for one week before experimentation in an air-conditioned atmosphere, at a temperature of 25°C, and with alternate light and dark cycles. In addition, a standard diet and water were provided ad libitum. Standard diet pellets contained not less than 20% protein, 5% fiber, 3.5% fat, 6.5% ash, and a vitamin mixture according to the standard guidelines.

### 2.4. Experimental Design

Rats were randomly classified into four groups (eight rats per group) and were injected as follows: (1) control group—nonirradiated rats injected with 0.5% DMSO in normal saline (0.5 ml/100 g BW IP) for 5 days; (2) irradiated saline-injected rats—rats injected with 0.5% DMSO in normal saline (0.5 ml/100 g BW IP) on day 1 and exposed to 3.2 Gy whole-body gamma radiations on day 3; (3) irradiated carvacrol-injected rats—rats injected with CAR in 0.5% DMSO in normal saline (80 mg/kg BW IP) for 5 days, 3 days before exposure to whole-body irradiation (3.2 Gy) and 2 days after; and (4) irradiated thymol-injected rats—rats injected with a single dose of thymol in 0.5% DMSO in normal saline (50 mg/kg BW IP) for 5 days, 3 days before exposure to whole-body irradiation (3.2 Gy) and 2 days after. Carvacrol-thymol doses were chosen according to previous studies, respectively [32–34]. Rats were weighed daily until the day of sacrifice. Two days post irradiation, six rats in each group were utilized for biological assessment and three rats in each group were utilized for histopathology and immunohistochemistry studies. After the experimental period (5 days), rats were fasted for 12 hours, subjected to a gradually increasing concentration of CO_2_, and sacrificed by decapitation. Blood sample was collected and allowed to clot, and ovarian tissues were dissected, washed with ice-cold saline, and weighed.

#### 2.4.1. Tissue Collection and Processing

Serum was separated by centrifugation at 3000 g for 15 min and kept frozen at -80°C until used in the assessment of anti-Mullerian hormone (AMH), estradiol (E2), follicle-stimulating hormone (FSH), and IGF-1 levels. Ovarian samples were homogenized at 1 : 10 (*w* : *v*) in the available KIT lysis buffer with an Ultra-Turrax Homogenizer. Then, the supernatant was obtained by centrifugation at 10,000 g for 15 minutes and kept at -80°C until the assessment of total antioxidant capacity (TAC) and TNF-*α* inflammatory marker activity. In addition, three ovaries from each group were fixed in an appropriate buffer for histopathological examination and morphometric analysis as well as for the immunohistochemical detection of the proliferating marker, proliferating cell nuclear antigen (PCNA).

#### 2.4.2. Histopathological Examination

Ovaries were fixed in 10% neutral-buffered formalin. After fixation, specimens were dehydrated in an ascending series of alcohols, cleared in two changes of xylene and embedded in molten paraffin. Sections of 4-micron thickness were cut using a rotary microtome and mounted on clean slides. For light microscopic histological examination, sections were stained with hematoxylin and eosin (H&E) [35]. The fifth cut was chosen to determine the antral follicular count (AFC) and to evaluate follicular development using a digital video camera mounted on a light microscope (DX 72, Olympus, Japan). We have used the most suitable methods for classifying follicles, which depend on their follicular development [36]. Atretic follicles were identified due to the presence of degenerating oocyte or pyknotic granulosa cells [37].

#### 2.4.3. Assessment of Circulating Levels of AMH, Estradiol, and FSH

To provide an accurate assessment of the follicular reserve as related to functional maturation of the ovary, serum AMH was assayed during the prepubertal period using the AMH ELISA Kit (Uscn Life Science Inc., Wuhan). Furthermore, serum estradiol and FSH were measured using chemiluminescent assay kits (Abcam, USA and IBL International GMBH, Germany, respectively).

#### 2.4.4. Assessment of Serum IGF-1 Levels

Due to its important role in follicular growth and in the ovulation process, serum IGF-1 was assessed using a commercially available kit, the Quantikine Rat/Mouse IGF-1 Immunoassay Kit (R&D Systems Inc.). According to the manufacturer's instructions, the intensity of the yellow color that was measured at 450 nm is in proportion to serum IGF-1 levels and expressed as ng/ml.

#### 2.4.5. Assessment of Oxidative Stress and Inflammatory Markers in Ovarian Tissue

TAC was measured in ovarian homogenates according to the manufacturer's instructions using a Randox assay kit (Randox Laboratories Ltd., Crumlin, UK). Moreover, TNF-*α* was assessed using a commercially available kit, the Rat TNF-*α* ELISA Kit (R&D Systems Inc.). The level of TNF-*α* was directly proportional to the color reaction and was expressed as pg/gm wet tissue.

#### 2.4.6. Immunohistochemistry

The immunohistochemistry protocol was carried out on 5 *μ*m thickness paraffin sections of the corresponding blocks using the streptavidin-biotinylated horseradish peroxidase (S-ABC) method (Novalink Max Polymer detection system, Novocastra, product no. RE7280-K). Endogenous peroxidase activity was inhibited by 3% H_2_O_2_ in distilled water for 5 min, and then the sections were washed in Tris-buffered saline (TRS) (Sigma-Aldrich, T 5030-100 TAB, pH 7.6) twice; 5 min for each. Nonspecific binding to antibodies was blocked by incubation with a protein block for 5 min (Novocastra). After the primary antibodies were incubated and washed, slides were incubated with biotinylated anti-mouse IgG (Novocastra) for 30 min. Finally, peroxidase was detected with a working solution of diaminobenzidine (DAB) substrate (Novocastra), and sections were washed, counterstained with Mayer's hematoxylin, and then mounted in DPX (dysterene, plasticizer, and xylene).

*2.4.6.1. Proliferative Marker*. The immunohistochemical analysis of PCNA [38] was carried out using a mouse anti-PCNA polyclonal-anti-mouse. The ab-2426 primary antibody was used for performing the streptavidin-biotinylated horseradish peroxidase (S-ABC) method (Novalink Max Polymer detection system, Novocastra, product NO. RE7280-K); the secondary antibody used was the biotinylated anti-mouse IgG (Novocastra). For negative control sections, the same procedure was followed but with the omission of incubation with the primary antibodies. To obtain an estimate of the percentage of proliferating cells, the percentage of nuclei positively stained for PCNA from the total number of granulosa cells was estimated in six high-power fields (40×) using a digital video camera.

#### 2.4.7. Statistical Analysis

Data are presented as mean ± SEM. Multiple comparisons were performed using one-way ANOVA followed by the Tukey-Kramer as a post hoc test. The 0.05 level of probability was used as the criterion for significance. All statistical analyses were performed and graphs were sketched using GraphPad Prism (ISI® software, USA) version 5 software.

## 3. Results

### 3.1. Ovarian Weight and Relative Ovarian Weight Changes

Two days after irradiation, rat ovaries weighed less than the control group by about 0.52-fold as shown in Table 1. In addition, the relative ovary weight was significantly lowered by 0.48-fold as compared to the control group. However, concurrent treatment with CAR or thymol increased the weight gained by ovaries as well as their relative ovary weight when compared with the irradiated group (Table 1).

### 3.2. Ovarian Histology and Morphometric Analysis

**Figure 1:**
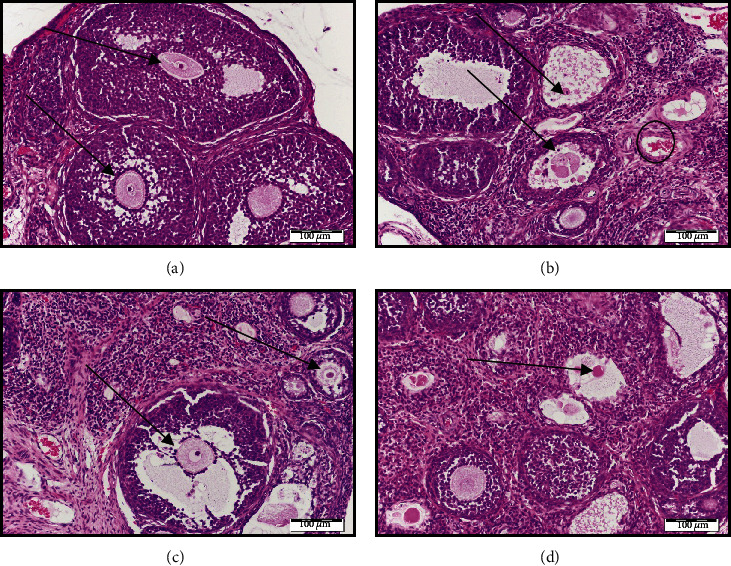
Photomicrographs of ovarian sections stained with hematoxylin and eosin. (a) Histological sections of the control ovaries showing the normal histopathological structure with multiple follicles of different stages (arrows), intact oocytes (O), and granulosa cells (g). (b) *γ*-Irradiated ovarian sections show few follicles with hemorrhage in the cortex (circle). Many small primary follicles are atretic (a) with degenerating oocytes and granulosa cells in irradiated ovaries (arrows). (c and d) Carvacrol/thymol-*γ*-irradiated ovarian sections showing similar organization as the control group. Scale bar, 20 *μ*m. gf: Graffian follicle, S: stroma.

Ovarian sections of the control group stained with H&E showed a normal histological structure of the cortex and medulla and multiple growing and mature follicles with normal layers of granulosa cells, normal liquor, and oocyte with clear zona pellucida, cytoplasm, nucleus, and nucleolus (arrows) as shown in Figure 1(a). On the other hand, irradiated ovaries showed marked shrinkage of the ovary and most of the follicles are degenerated with degenerated oocytes (arrows) as shown in Figure 1(b). CAR-irradiated ovaries show moderate restoration of the ovarian size with the appearance of normal oocytes, while there are still some degenerated follicles (arrows) (Figure 1(c)). However, the thymol-irradiated group showed the restoration of the volume of the ovary with a complete amelioration of the radiation-induced ovarian follicular loss (arrows) (Figure 1(d)).

Recently, it was reported that the healthy antral follicular count (AFC) serves as a marker of ovarian reserve and correlates with serum AMH levels [39, 40]. In the current study, irradiated ovaries showed a significant reduction in the AFC reaching about 0.13-fold as compared with the control group, while the number of atretic follicles significantly increased by 4-fold as compared with the control group. However, concurrent treatment with CAR and thymol significantly increased the healthy AFC to 4- and 5-fold of the irradiated group and reduced the atretic follicles to 0.42- and 0.58-fold of the irradiated group.

### 3.3. Serum Hormone Levels

**Figure 2:**
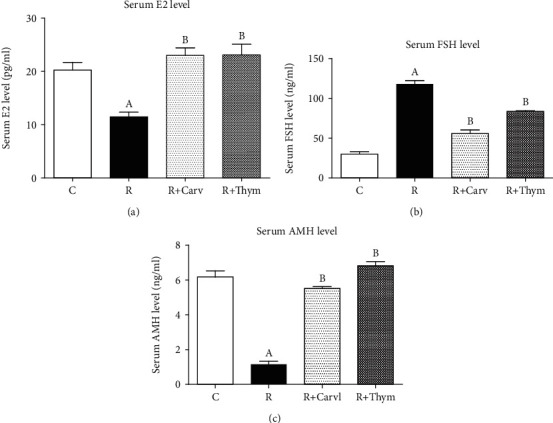
Changes in serum AMH—anti-Mullerian hormone (a), FSH—follicular stimulating hormone (b), and E2—estradiol (c) expressed as pg/ml, following carvacrol or thymol treatment in rats subjected to *γ*-irradiation. Data are given as mean ± SEM for groups of 5 rats. A or B: statistically significant from the control or radiation group, respectively, at *P* < 0.05 using one-way analysis of variance (ANOVA) followed by the Tukey-Kramer as a post hoc test.

It has been found recently that AMH is a measure for the primordial pool, another indicator of POF [41]. Two days post irradiation, rats had very low levels of serum AMH and E2 (1.15 ± 0.17*vs.*6.17 ± 0.36 ng/ml in controls) reaching about 0.19- and 0.56-fold of the control values, respectively. However, irradiated rats that received CAR or thymol had normal levels of both AMH and E2 when compared to the control group as shown in Figures 2(a) and 2(b). In addition, Figure 2(c) shows a marked increase in serum FSH level (118.00 ± 3.13*vs.*29.85 ± 3.13 ng/ml in controls) two days post irradiation reaching 3.95-fold as compared to the control group. In contrast, CAR and thymol-irradiated rats showed a significant reduction in serum FSH levels as compared with the irradiated group; however, they are still higher than the control group as shown in Figure 2(c).

### 3.4. Proliferation Marker

**Figure 3:**
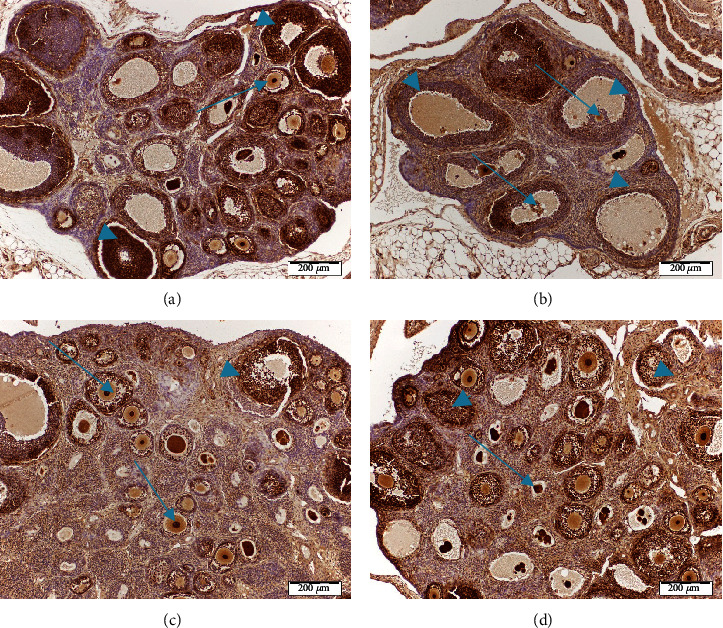
Immunohistochemical localization of PCNA in ovarian follicles was studied 4 days after irradiation. (a) Expression of PCNA in ovaries of the control group showed a high degree of PCNA expression in the oocyte and granulosa cells of growing follicles (brown color). (b) Expression of PCNA in the ovaries of rats subjected to *γ*-radiation (3.2 Gy) showed a decreased expression with O and GC. (c and d) Expression of PCNA in the ovaries of rats treated with carvacrol or thymol and exposed to *γ*-radiation (c) or showed a high PCNA expression of oocyte and granulosa cells of all growing follicles (brown color) (d). Scale bar, 20 *μ*m. GC: granulosa cells, O: oocyte.

Besides morphometric analysis, immunohistochemical detection of the proliferation marker, PCNA, proved that follicles belonging to the same follicular pool were at different states of maturation in control and irradiated ovaries as shown in Figure 3. Ovarian sections showed strong nuclear immunostaining for PCNA in all oocytes and proliferating granulosa cells of the growing follicles (primary (arrow) and secondary follicles (arrowheads)) in almost all groups except for the irradiated one (Figures 3(a), 3(c), and 3(d)). In contrast, irradiated ovaries showed a marked reduction in the immune reaction of the secondary follicles of granulosa cells (arrowheads) and very weak immune positivity (arrows) in the nuclei of their degenerated oocytes except for radiation-resistant late-antral follicles which stained positive. For different groups, atretic follicles undergoing atresia were negative for PCNA as previously reported [42].

### 3.5. Oxidative Stress Markers

Gamma-irradiation-induced oxidative stress in rat ovaries was assessed by the determination of TAC. As shown in Table 1, radiation caused a significant depletion of the antioxidant activity reaching about 87% of the control group. Treatment with CAR/thymol significantly improved these levels to 117% and 115% as compared to the irradiated group, respectively (Table 1).

### 3.6. Correlation between TNF-*α* and IGF-1 Levels

**Figure 4:**
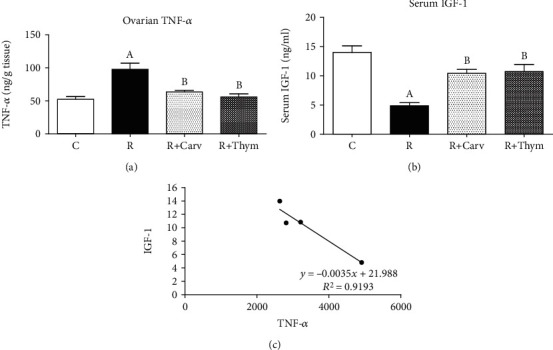
(a) Changes in ovarian TNF-*α* expressed as pg/ml, following carvacrol or thymol treatment in rats subjected to *γ*-irradiation. (b) Changes in serum IGF-1 following carvacrol or thymol treatment in rats subjected to *γ*-irradiation, expressed as ng/ml. (c) Quantitative correlation between ovarian TNF-*α* and serum IGF-1. Data are given as Mean ± SEM for groups of 5 rats. A or B: statistically significant from the control or radiation group, respectively, at *P* < 0.05 using one-way analysis of variance (ANOVA) followed by the Tukey-Kramer as a post hoc test.

In order to complete our assessment of the cross-talk between TNF-*α* and IGF-1 levels, ovarian TNF-*α* and serum IGF-1 were determined calorimetrically. Two days post irradiation, results showed a marked increase in ovarian TNF-*α* activity by around 1.87-fold which was associated with a 50% decrease in serum IGF-1 as compared with the control values. On the other hand, CAR and thymol treatment significantly rectified this inverse relationship between TNF-*α* and IGF-1 reaching reductions of about 34.67% and 42.86% in TNF-*α* activity (Figure 4(a)) at 214% and 220% increments in IGF-1 levels as compared to the irradiated group, respectively (Figure 4(b)). Correlation was quantitatively shown in Figure 4(c).

## 4. Discussion

Increased cancer survival rates in females have raised the immense need for long-term protection against toxicities of cancer therapy [5, 6]. Although some strategies have been used recently, such as oocyte and ovarian tissue cryopreservation for female fertility preservation [43, 44], cytotoxic damage to ovarian stromal and germ cells appears to be progressive and irreversible [43]. In addition, these measures either underuse or require invasive techniques for the collection of ovarian tissues as well as for autologous transplantation [44]. Therefore, the development of simpler pharmacological method is of clinical importance. Plant-derived chemopreventive agents exhibit limited side effects and less toxicity and at the same time protect the normal cells against radiation [21]. Recently, Arivalagan et al. [45] have documented the radioprotective effect of CAR against X-radiation in cultured human blood lymphocytes. In addition, a study by Archana et al. has clearly documented the antioxidant, anticlastogenic, and radioprotective potentials of thymol in gamma-irradiated mice [46]. However, both studies solely attributed this radioprotection to the normalization of intracellular antioxidant levels and the cellular mechanisms have not been fully investigated [45, 46]. Furthermore, no study has explored their potential radioprotective role on the female ovarian follicular loss induced by gamma irradiation. Therefore, the present study was aimed at exploring the molecular mechanisms underlying the prospective radioprotective effect of CAR and thymol and compare between them.

Patients with POF have elevated levels of FSH and E2 (cycle day 3) and considerably lower levels of AMH, as well as low AFC levels which have been used as markers of ovarian failure [39]. In this study, gamma radiation induced a typical POF which was manifested in rats as low serum E2 and high FSH levels when compared with the control values; however, both CAR and thymol significantly improved the hormonal changes induced by irradiation. A previous study reported that *S. khuzestanica* essential oil (SKEO), in which CAR is one of the main constituents, significantly increased the E2 levels in busulfan-induced ovarian failure [47]. However, no studies have been found to demonstrate or compare the protective effect of CAR and thymol in experimentally induced ovarian failure rats. Accordingly, this study is the first to find that CAR is superior over thymol in its effect on the FSH level, while they have the same impact with regard to the E2 level.

Besides the FSH and E2 assessment, AMH was measured in different groups as it is considered the most sensitive tool to determine the ovarian reserve before infertility develops and to predict the ovarian failure induced by cytotoxic therapy [39, 48]. In this study, irradiated rats showed a minute level of serum AMH and lower AFC as compared with the control level; these defects were improved in the irradiated rats treated with CAR or thymol. This study showed a correlation between AMH level reduction and FSH level increment, which reflects the failure of the ovarian function as a result of exposure to irradiation. Moreover, our present results show that thymol has a superior ovarian reserve protection over the CAR effect. However, administration of both CAR or thymol alone increased the AMH levels when compared to the control rats (data not shown), a result which sheds lights on the potential ovarian preservation effect of these phenolic compounds in other forms of ovarian insufficiency.

Besides, PCNA is involved in follicular growth and its expression increases during the gonadotropin-dependent stages of preovulatory follicular development [49, 50]. Therefore, the expression of PCNA found a significant reduction in the proliferation of granulosa cells of irradiated ovaries. This could be explained as *γ*-irradiation induces apoptosis through a p21-mediated mechanism which directly inhibits PCNA in DNA replication [51]. Additionally, in control and treated groups, the expression of PCNA coincided with the initiation of follicle growth which was in agreement with previous studies [52]. However, PCNA immunoreactivity was present only in oocytes of primordial follicles with stain-free pregranulosa cells. These findings were in accordance with our previous study which suggested a role for this protein even in the earlier stages of folliculogenesis [53].

It has been long recognized that the damaging effects of ionizing radiation is brought about by direct DNA ionization as well as indirectly through ROS production. As a consequence, thiols like GSH and other antioxidant enzymes compete with this oxidation and repair the damage [54]. Previous findings have confirmed the protective effects and usefulness of antioxidants in infertility and reproduction system disorders [55]. Both essential oils restored the ovarian total antioxidant activity after a single dose of *γ*-irradiation. In this context, a study has shown that SKEO, an Iranian plant that contains CAR, improved the fertility disorders induced by busulfan in female rats due to its antioxidative effect [47] and both phenolic compounds suppressed doxorubicin-induced oxidative stress in rat brains [56]. Our study highlighted that blocking oxidative stress by these compounds was observed in rats rescued from the *γ*-irradiation-induced ovarian failure; thus, we are the first to prove that these essential oils rescued the ovary through their antioxidative mechanism.

In the context of the deleterious effects of irradiation, another pathway involving inflammation has been identified in which numerous proinflammatory cytokines and chemokines are excessively produced immediately following exposure such as interleukin-1 (IL-1), IL-6, and TNF-*α* [57]. Moreover, the best example of the relationship between ovulatory infertility and inflammation is premature ovarian failure (POF) [58]. Some pharmacological measures preserved the radiation-induced ovarian failure through their anti-inflammatory mechanisms such as zingerone [59], curcumin, and capsaicin [60]. Both CAR and thymol have been found to possess significant health benefits as anticancer and chemopreventive agents [23, 27, 61] possibly through the following anti-inflammatory effects: inhibition of TNF-*α* [61] and downregulation of NF-*κ*B and MAPK signaling pathways [29]. In the present study, concomitant treatment of irradiated rats with CAR or thymol has significantly inhibited TNF-*α* in the downstream signaling pathway of inflammation induced by irradiation. Therefore, another mechanism by which these compounds enhance the in vivo follicular development could be a result of decreasing irradiation-mediated inflammatory signals.

It is well known that IGF-1 and the IGF-1 receptor have an important cytoprotective role against radiation-induced ovarian damage [40, 62]. Accordingly, it was interesting to assess the serum IGF-1 levels of different treatment groups and it was found that there was a significant reduction in the serum IGF-1 level two days post irradiation; this could be reversed with the administration of CAR and thymol.

Finally, this study is the first to explore the effect of CAR/thymol on the cross-talk between TNF-*α* and IGF-1 in radiotherapy-induced ovarian damage. This cross-talk could control the inflammatory processes as well as the decision concerning apoptosis or cell survival [63]. Our current study showed a correlated inverse relationship between TNF-*α* and IGF-1 in irradiated rats; this was in accordance with a previous study which found that Zymosan increased TNF-*α*, and this was associated with a 40% decrease in the IGF-I concentration in plasma, liver, heart, and brain [64]. Treatment with CAR or thymol rectified this relationship and significantly counteracted the irradiation-induced suppression of IGF-1. In this context, the present study suggests a novel mechanism for CAR and thymol radioprotection which might be through the modulation of the cross-talk between TNF-*α* and IGF-1 levels as well as the potentiation of IGF-1-mediated antioxidant and cytoprotection effects.

However, this rat model only offers information about the protection of carvacrol or thymol on the short-term negative impact of radiation, but investigations of longer-term effects on the primordial pool after irradiation is becoming necessary before advancing into human trials.

## 5. Conclusions

The present study demonstrates that CAR and thymol rescue the ovarian reserve and enlighten new radioprotection mechanisms against the deleterious effects of gamma irradiation on ovaries. These mechanisms could be through the preservation of AMH and granulosa cell proliferation and decreasing oxidative stress and inflammatory pathways. In addition, our study is the first to suggest the promising role of TNF-*α* and IGF-1 cross-talk in the radioprotective effect of CAR and thymol. Finally, our findings clearly warrant the potential clinical applications of these phenolic compounds in infertile females with poor ovarian reserve.

## Figures and Tables

**Table 1:** Effect of CAR or thymol (80 mg/kg IP; once daily for 5 days, respectively) on ovarian weight, relative ovary weight, and total antioxidant capacity in rats subjected to a single dose of whole-body irradiation (3.2 Gy).

Treated groups	Ovarian weight (mg)	Relative ovary wt (mg/100 g body weight)	TAC (*μ*M/l)
Control	39.25 ± 3.20	87.24 ± 6.2	37.6 ± 1.2
IR	16.53 ± 0.66^**a**^	41.33 ± 0.7^**a**^	32.8 ± 0.65
IR/CAR	28.25 ± 2.51^**a**,**b**^	62.78 ± 4.69^**b**^	38.6 ± 0.8
IR/thymol	26.91 ± 3.10^**a**,**b**^	58.49 ± 1.3^**b**^	38.2 ± 1.2

Data expressed as mean ± SEM (*n* = 6). ^a,^^b^Significantly different from the control or radiation group, respectively, at *P* < 0.05 using one-way ANOVA followed by the Tukey-Kramer as a post hoc test. IR: irradiation; IR/CAR: irradiation/carvacrol; CAR: carvacrol; TAC: total antioxidant capacity.

## Data Availability

All data used to support the findings of this study are included within this article.

## Abbreviations

POF: Premature ovarian failure
CAR: Carvacrol
IGF-1: Insulin-like growth factor-1
TNF-*α*: Tumor necrosis factor-alpha
PCNA: Proliferating cell nuclear antigen
AMH: Anti-Mullerian hormone
FSH: Follicle-stimulating hormone
TAC: Total antioxidant capacity
AFC: Antral follicular count
TRS: Tris-buffered saline.

## References

[1] Joiner M. C., van der Kogel A. J., Steel G. G., Joiner M. C. A. K. (2009). Introduction: the significance of radiobiology and radiotherapy for cancer treatment. *Basic Clinical Radiobiology*.

[2] Duncan F. E., Kimler B. F., Briley S. M. (2016). Combating radiation therapy-induced damage to the ovarian environment. *Future Oncology*.

[3] Stroud J. S., Mutch D., Rader J., Powell M., Thaker P. H., Grigsby P. W. (2009). Effects of cancer treatment on ovarian function. *Fertility and Sterility*.

[4] Wallace W. H. B., Thomson A. B., Saran F., Kelsey T. W. (2005). Predicting age of ovarian failure after radiation to a field that includes the ovaries. *International Journal of Radiation Oncology, Biology, Physics*.

[5] Oktay K., Harvey B. E., Partridge A. H. (2018). Fertility preservation in patients with cancer: ASCO clinical practice guideline update. *Journal of Clinical Oncology*.

[6] Imai A., Furui T., Yamamoto A. (2008). Preservation of female fertility during cancer treatment. *Reproductive Medicine and Biology*.

[7] Adriaens I., Smitz J., Jacquet P. (2009). The current knowledge on radiosensitivity of ovarian follicle development stages. *Human Reproduction Update*.

[8] Meirow D., Biederman H., Anderson R. A., Wallace W. H. B. (2010). Toxicity of chemotherapy and radiation on female reproduction. *Clinical Obstetrics and Gynecology*.

[9] Buyuk E., Nejat E., Neal-Perry G. (2010). Determinants of female reproductive senescence: differential roles for the ovary and the neuroendocrine axis. *Seminars in Reproductive Medicine*.

[10] Gosden R. G., Wade J. C., Fraser H. M., Sandow J., Faddy M. J. (1997). Impact of congenital or experimental hypogonadotrophism on the radiation sensitivity of the mouse ovary. *Human Reproduction*.

[11] Wallace W. H. B., Thomson A. B., Kelsey T. W. (2003). The radiosensitivity of the human oocyte. *Human Reproduction*.

[12] Borek C. (2004). Antioxidants and radiation therapy. *The Journal of Nutrition*.

[13] Fawcett S. L., Gomez A. C., Barter S. J., Ditchfield M., Set P. (2012). More harm than good? The anatomy of misguided shielding of the ovaries. *The British Journal of Radiology*.

[14] Irtan S., Orbach D., Helfre S., Sarnacki S. (2013). Ovarian transposition in prepubescent and adolescent girls with cancer. *The Lancet Oncology*.

[15] Hofer M., Hoferová Z., Falk M. (2017). Pharmacological modulation of radiation damage. Does it exist a chance for other substances than hematopoietic growth factors and cytokines?. *International Journal of Molecular Sciences*.

[16] Behl R., Kaul R. (2002). Insulin like growth factor 1 and regulation of ovarian function in mammals. *Indian Journal of Experimental Biology*.

[17] Giudice L. C. (1992). Insulin-like growth factors and ovarian follicular development. *Endocrine Reviews*.

[18] Chun S. Y., Billig H., Tilly J. L., Furuta I., Tsafriri A., Hsueh A. J. (1994). Gonadotropin suppression of apoptosis in cultured preovulatory follicles: mediatory role of endogenous insulin-like growth factor I. *Endocrinology*.

[19] Wang M., Tsai B., Brown J. W., Meldrum D. R. (2003). Insulin-like growth factor-1 in myocardial tissue: interaction with tumor necrosis factor. *Critical Care*.

[20] Anwar A., Zahid A. A., Scheidegger K. J., Brink M., Delafontaine P. (2002). Tumor necrosis factor-*α* regulates insulin-like growth factor-1 and insulin-like growth factor binding protein-3 expression in vascular smooth muscle. *Circulation*.

[21] Arivalagan S., Thomas N. S., Chandrasekaran B. (2015). Combined therapeutic efficacy of carvacrol and X-radiation against 1,2-dimethyl hydrazine-induced experimental rat colon carcinogenesis. *Molecular and Cellular Biochemistry*.

[22] Akalin G., İncesu Z. (2011). The effects of carvacrol on apoptosis of H-ras and N-ras transformed cell lines. *Turkish Journal of Pharmaceutical Sciences*.

[23] Sökmen A., Sökmen M., Daferera D. (2004). The *in vitro* antioxidant and antimicrobial activities of the essential oil and methanol extracts of *Achillea biebersteini* Afan. (Asteraceae). *Phytotherapy Research*.

[24] Sivaranjani A., Sivagami G., Nalini N. (2016). Chemopreventive effect of carvacrol on 1,2-dimethylhydrazine induced experimental colon carcinogenesis. *Journal of Cancer Research and Therapeutics*.

[25] Kianmehr M., Rezaei A., Boskabady M. H. (2016). Effect of carvacrol on various cytokines genes expression in splenocytes of asthmatic mice. *Iranian Journal of Basic Medical Sciences*.

[26] Aristatile B., Al-Assaf A. H., Pugalendi K. V. (2013). Carvacrol suppresses the expression of inflammatory marker genes in D-galactosamine-hepatotoxic rats. *Asian Pacific Journal of Tropical Medicine*.

[27] Tsai M.-L., Lin C.-C., Lin W.-C., Yang C.-H. (2011). Antimicrobial, antioxidant, and anti-inflammatory activities of essential oils from five selected herbs. *Bioscience, Biotechnology, and Biochemistry*.

[28] Jafari A., Rasmi Y., Hajaghazadeh M., Karimipour M. (2018). Hepatoprotective effect of thymol against subchronic toxicity of titanium dioxide nanoparticles: biochemical and histological evidences. *Environmental Toxicology and Pharmacology*.

[29] Liang D., Li F., Fu Y. (2014). Thymol inhibits LPS-stimulated inflammatory response via down-regulation of NF-*κ*B and MAPK signaling pathways in mouse mammary epithelial cells. *Inflammation*.

[30] Abedi S. M., Yarmand F., Motallebnejad M. (2016). Radioprotective effect of thymol against salivary glands dysfunction induced by ionizing radiation in rats. *Iranian Journal of Pharmaceutical Research: IJPR*.

[31] Lee Y. K., Chang H. H., Kim W. R., Kim J. K., Yoon Y. D. (1998). Effects of gamma-radiation on ovarian follicles. *Arhiv za higijenu rada i toksikologiju*.

[32] Ozen B. D., Uyanoglu M. (2018). Effect of carvacrol on IL-6/STAT3 pathway after partial hepatectomy in rat liver. *Bratislava Medical Journal*.

[33] Türkcü G., Alabalık U., Keleş A. N. (2015). Protective effects of carvacrol and pomegranate against methotrexate-induced intestinal damage in rats. *International Journal of Clinical and Experimental Medicine*.

[34] Koc K., Cerig S., Ucar S. (2018). Gastroprotective effects of oleuropein and thymol on indomethacin-induced gastric ulcer in Sprague-Dawley rats. *Drug and Chemical Toxicology*.

[35] Banchroft J., Stevens A., Turner D. (1996). *Theory and practice of histological techniques*.

[36] Britt K. L., Drummond A. E., Cox V. A. (2000). An age-related ovarian phenotype in mice with targeted disruption of the *Cyp 19* (aromatase) gene. *Endocrinology*.

[37] Braw R. H., Tsafriri A. (1980). Effect of PMSG on follicular atresia in the immature rat ovary. *Reproduction*.

[38] Oktay K., Schenken R. S., Nelson J. F. (1995). Proliferating cell nuclear antigen marks the initiation of follicular growth in the rat. *Biology of Reproduction*.

[39] Jankowska K. (2017). Premature ovarian failure. *Przegla̜d Menopauzalny = Menopause Review*.

[40] Mahran Y. F., El-Demerdash E., Nada A. S., El-Naga R. N., Ali A. A., Abdel-Naim A. B. (2015). Growth hormone ameliorates the radiotherapy-induced ovarian follicular loss in rats: impact on oxidative stress, apoptosis and IGF-1/IGF-1R axis. *PLoS ONE*.

[41] Kim H., Yamanouchi K., Nishihara M. (2006). Expression of ski in the granulosa cells of atretic follicles in the rat ovary. *Journal of Reproduction and Development*.

[42] Sonmezer M., Oktay K. (2006). Fertility preservation in young women undergoing breast cancer therapy. *The Oncologist*.

[43] Imai A., Ichigo S., Matsunami K., Takagi H., Kawabata I. (2017). Ovarian function following targeted anti-angiogenic therapy with bevacizumab. *Molecular and Clinical Oncology*.

[44] Han S. S., Kim Y. H., Lee S. H. (2011). Underuse of ovarian transposition in reproductive-aged cancer patients treated by primary or adjuvant pelvic irradiation. *Journal of Obstetrics and Gynaecology Research*.

[45] Arivalagan S., Thomas N. S., Kuppusamy T., Namashivayam N. (2015). Radioprotective effect of carvacrol against X-radiation-induced cellular damage in cultured human peripheral blood lymphocytes. *Journal of Environmental Pathology, Toxicology and Oncology*.

[46] Archana P. R., Rao B. N., Rao B. S. S. (2011). *In vivo* radioprotective potential of thymol, a monoterpene phenol derivative of cymene. *Mutation Research/Genetic Toxicology and Environmental Mutagenesis*.

[47] Ahmadi A., Chafjiri S. B., Sadrkhanlou R. A. (2017). Effect of *Satureja khuzestanica* essential oil against fertility disorders induced by busulfan in female mice. *Veterinary Research Forum*.

[48] Krawczuk-Rybak M., Leszczynska E., Poznanska M., Zelazowska-Rutkowska B., Wysocka J. (2013). Anti-müllerian hormone as a sensitive marker of ovarian function in young cancer survivors. *International Journal of Endocrinology*.

[49] Maruo T., Laoag-Fernandez J. B., Takekida S. (1999). Regulation of granulosa cell proliferation and apoptosis during follicular development. *Gynecological Endocrinology*.

[50] Salvetti N. R., Stangaferro M. L., Palomar M. M. (2010). Cell proliferation and survival mechanisms underlying the abnormal persistence of follicular cysts in bovines with cystic ovarian disease induced by ACTH. *Animal Reproduction Science*.

[51] Waga S., Hannon G. J., Beach D., Stillman B. (1994). The p21 inhibitor of cyclin-dependent kinases controls DNA replication by interaction with PCNA. *Nature*.

[52] Xu B., Hua J., Zhang Y. (2011). Proliferating cell nuclear antigen (PCNA) regulates primordial follicle assembly by promoting apoptosis of oocytes in fetal and neonatal mouse ovaries. *PLoS ONE*.

[53] Mahran Y. F., El-Demerdash E., Nada A. S., Ali A. A., Abdel-Naim A. B. (2013). Insights into the protective mechanisms of tamoxifen in radiotherapy-induced ovarian follicular loss: impact on insulin-like growth factor 1. *Endocrinology*.

[54] Navarro J., Obrador E., Pellicer J. A., Asensi M., Viña J., Estrela J. M. (1997). Blood glutathione as an index of radiation-induced oxidative stress in mice and humans. *Free Radical Biology and Medicine*.

[55] Safarnavadeh T., Rastegarpanah M. (2011). Antioxidants and infertility treatment, the role of *Satureja khuzestanica*: a mini-systematic review. *Iranian Journal of Reproductive Medicine*.

[56] Mohebbati R., Jalili-Nik M., Paseban M., Shafei M. N., Khajavirad Rad A. (2018). Effects of *Zataria multiflora* extract and carvacrol on doxorubicin-induced oxidative stress in rat brain. *Pharmaceutical Sciences*.

[57] Kim J. H., Jenrow K. A., Brown S. L. (2014). Mechanisms of radiation-induced normal tissue toxicity and implications for future clinical trials. *Radiation Oncology Journal*.

[58] Weiss G., Goldsmith L. T., Taylor R. N., Bellet D., Taylor H. S. (2009). Inflammation in reproductive disorders. *Reproductive Sciences*.

[59] Kaygusuzoglu E., Caglayan C., Kandemir F. M. (2018). Zingerone ameliorates cisplatin‐induced ovarian and uterine toxicity via suppression of sex hormone imbalances, oxidative stress, inflammation and apoptosis in female Wistar rats. *Biomedicine & Pharmacotherapy*.

[60] Melekoglu R., Ciftci O., Eraslan S., Cetin A., Basak N. (2018). Beneficial effects of curcumin and capsaicin on cyclophosphamide-induced premature ovarian failure in a rat model. *Journal of Ovarian Research*.

[61] Yao L., Hou G., Wang L., Zuo X. S., Liu Z. (2018). Protective effects of thymol on LPS-induced acute lung injury in mice. *Microbial Pathogenesis*.

[62] Floratou K., Giannopoulou E., Antonacopoulou A. (2012). Oxidative stress due to radiation in CD34^+^ hematopoietic progenitor cells: protection by IGF-1. *Journal of Radiation Research*.

[63] Vallée S., Fouchier F., Brémond P., Briand C., Marvaldi J., Champion S. (2003). Insulin-like growth factor-1 downregulates nuclear factor *κ*B activation and upregulates interleukin-8 gene expression induced by tumor necrosis factor *α*. *Biochemical and Biophysical Research Communications*.

[64] Fan J., Li Y. H., Bagby G. J., Lang C. H. (1995). Modulation of inflammation-induced changes in insulin-like growth factor (IGF)-I and IGF binding protein-1 by anti-TNF antibody. *Shock*.

